# Engineering acyl carrier protein to enhance production of shortened fatty acids

**DOI:** 10.1186/s13068-016-0430-4

**Published:** 2016-02-02

**Authors:** Xueliang Liu, Wade M. Hicks, Pamela A. Silver, Jeffrey C. Way

**Affiliations:** Wyss Institute for Biologically Inspired Engineering, CLSB Building 5th Floor, 3 Blackfan Circle, Boston, MA 02115 USA; School of Engineering and Applied Sciences, Harvard University, Cambridge, USA; Department of Systems Biology, Harvard Medical School, Boston, USA

**Keywords:** Acyl carrier protein, Protein engineering, Thioesterase, Free fatty acid, Lauric acid

## Abstract

**Background:**

The acyl carrier protein (ACP) is an essential and ubiquitous component of microbial synthesis of fatty acids, the natural precursor to biofuels. Natural fatty acids usually contain long chains of 16 or more carbon atoms. Shorter carbon chains, with increased fuel volatility, are desired for internal combustion engines. Engineering the length specificity of key proteins in fatty acid metabolism, such as ACP, may enable microbial synthesis of these shorter chain fatty acids.

**Results:**

We constructed a homology model of the *Synechococcus elongatus* ACP, showing a hydrophobic pocket harboring the growing acyl chain. Amino acids within the pocket were mutated to increase steric hindrance to the acyl chain. Certain mutant ACPs, when over-expressed in *Escherichia coli*, increased the proportion of shorter chain lipids; I75 W and I75Y showed the strongest effects. Expression of I75 W and I75Y mutant ACPs also increased production of lauric acid in *E. coli* that expressed the C12-specific acyl-ACP thioesterase from *Cuphea palustris*.

**Conclusions:**

We engineered the specificity of the ACP, an essential protein of fatty acid metabolism, to alter the *E. coli* lipid pool and enhance production of medium-chain fatty acids as biofuel precursors. These results indicate that modification of ACP itself could be combined with enzymes affecting length specificity in fatty acid synthesis to enhance production of commodity chemicals based on fatty acids.

**Electronic supplementary material:**

The online version of this article (doi:10.1186/s13068-016-0430-4) contains supplementary material, which is available to authorized users.

## Background

With the continuous rise in global energy needs and adverse climate changes, development of cleaner and renewable alternatives to fossil fuels has become paramount. Microbial synthesis of biofuels is an attractive, renewable alternative to fossil fuels [1–3]. Organisms naturally synthesize large quantities of fuel-like hydrocarbons in the form of lipids, which are used in cell membranes and other molecules. In microbes, the end products of fatty acid metabolism are long acyl chains consisting mostly of 16–18 carbons. When extracted for fuels, these long-chain carbon molecules remain solid at room temperature and lack favorable physical properties such as higher volatility and lower viscosity. Such properties are characteristic of medium-length (8–12) carbon chains used ubiquitously in fuels for vehicles and jets.

**Fig. 1:**
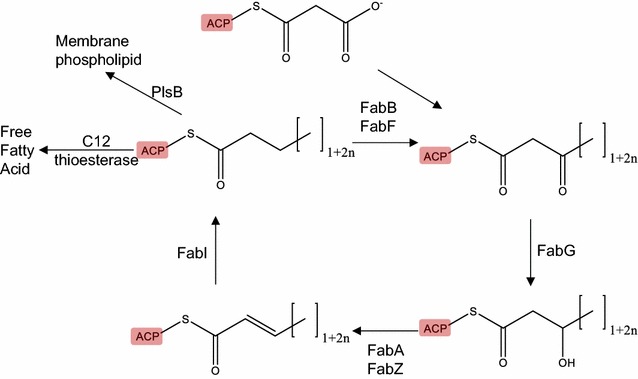
Overview of fatty acid synthesis. Fatty acid synthesis proceeds through iterative cycles of elongation. In each cycle, the acyl chain is extended by 2-carbons using a malonyl-ACP as a carbon donor (by FabB or FabF) and subsequently reduced into a saturated chain (by FabG, FabA, FabZ, and FabI). From the first 2-carbon malonyl-ACP to the final length fatty acid processed through this cycle, the hydrophobic acyl chain is attached to and shielded by the ACP instead of existing in a free form

**Fig. 2:**
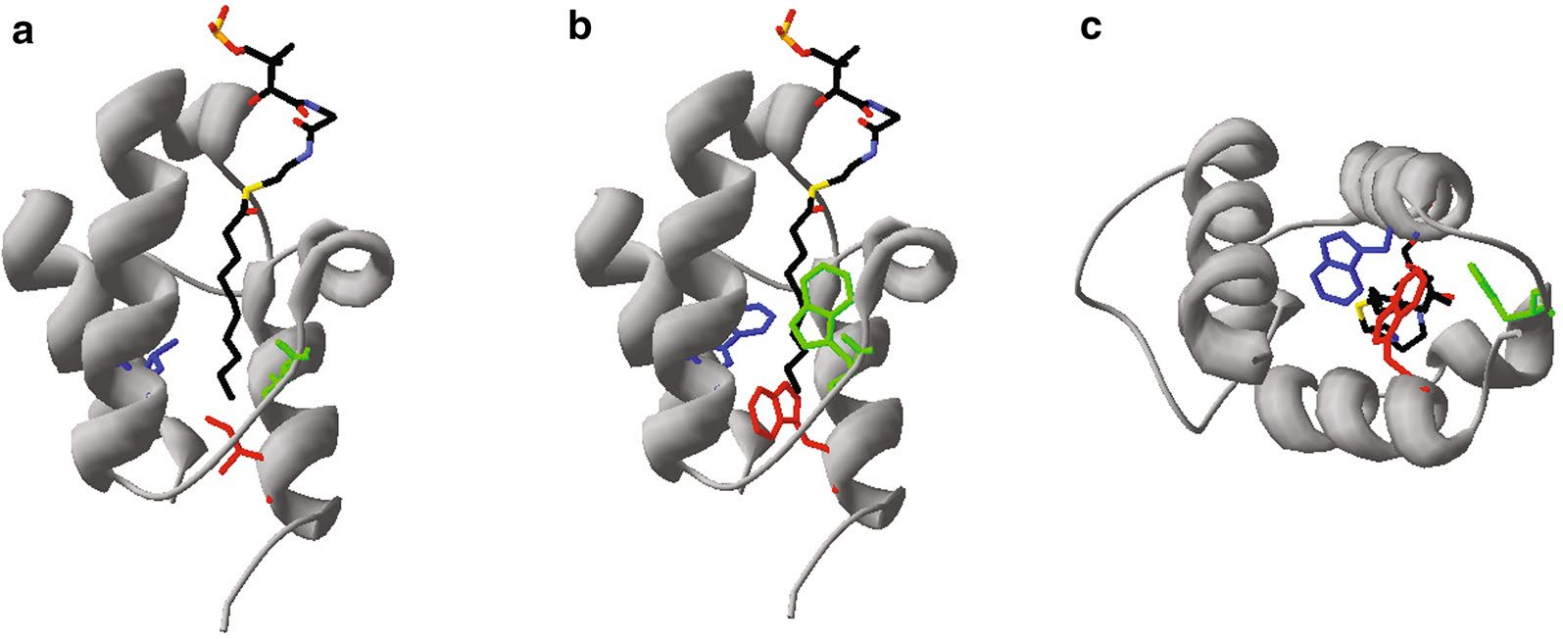
Se-ACP Structural homology models with WT and mutant residues. **a** Homology model of Se-ACP bound to a C10 acyl chain is shown. Highlighted in *blue* (residue 49), *green* (residue 57) and *red* (residue 75) are small hydrophobic amino acids lining the WT ACP pocket, Leu, Ile, and Ile, respectively. Each residue was separately mutated to a bulkier hydrophobic amino acid: methionine, tyrosine or tryptophan in order to induce steric hindrance and favor shorter chain fatty acid synthesis. **b** For illustration, a homology model with all three residues of interest mutated to tryptophan shows how each side chain might be positioned when mutated separately. Trp75 (*red*) extends closest to the acyl chain terminus. **c** Looking up through the axis of the acyl chain from the bottom perspective of the ACP pocket, Trp75 (*red*) is more directly in line with the acyl chain, as compared to the other mutant residues. This substitution appears to introduce direct steric hindrance to the acyl chain, while Trp at position 49 or 57 does not

Previous work on the biological synthesis of medium-length fuel precursors has employed thioesterase enzymes with medium-length chain specificity to release free fatty acids (FFA) from intermediates in fatty acid synthesis [4–7]. Here, we employ a complementary strategy to bias FFA synthesis toward shorter chains by engineering acyl carrier protein (ACP), an essential protein and key component of fatty acid metabolism. In fatty acid synthesis in bacteria and plants, ACP is attached to the acyl chain and presents it to the other enzymes during successive cycles of elongation and reduction (Fig. 1) [8–11]. ACP is a small (~9 kB), acidic (pI = 4.1) protein abundant in the cytoplasm, constituting about 0.25 % of all soluble proteins in *Escherichia coli* [8]. The structure of ACP is highly conserved even among variants with low sequence similarity. Four alpha helices, with the major helices I, II, and IV running parallel to each other, enclose a hydrophobic pocket that harbors the acyl chain; minor helix III runs perpendicular to these (Fig. 2). The acyl chain is connected to a 4-phosphopantetheine modification at a conserved serine and enters the hydrophobic cavity between helices II and III. Roujeinikova et al. solved the structures of *E. coli* ACP attached to C6, C7, and C10 fatty acids [12]. In each case, the distal end of the fatty acid terminates in a deep pocket within the protein near Ile72 (corresponding to Ile75 of the *Synechococcus elongatus* ACP), with the phosphopantetheine group also entering the pocket to varying degrees. Acyl chains up to eight carbons are fully bound within the pocket, with the thioester bond sequestered in the core of the protein [8, 12–14]. We therefore hypothesized that the size of ACP’s hydrophobic pocket influences the composition of lipid lengths in a cell. As the acyl chain grows to a length of around 16, the thioester bond becomes more fully solvent exposed, which may facilitate cleavage by downstream processing enzymes.

We found that over-expressing certain mutant ACPs altered the composition of the cellular lipid pool and increased production of certain medium-chain fatty acids. Our findings could be useful for microbial production of transportation biofuels based on metabolically engineered pathways.

## Results and discussion

To enhance production of medium-chain fatty acids, we constructed mutants of ACP designed to decrease the acyl chain pocket size (Fig. 2). Variants of the cyanobacterial (*S. elongatus*) ACP were expressed in an *E. coli* host. We chose *S. elongatus* ACP due to its potential compatibility with recently discovered enzymes of the cyanobacterial alkane biosynthesis pathway [15], which could enable microbial synthesis of fatty alcohol or alkanes. The native *E. coli* ACP gene was left intact, as we found that its knockout could not be rescued by complementation from expression of wild-type *E.coli* ACP encoded on a plasmid (data not shown). To determine which hydrophobic residues of *S. elongatus* ACP lined the inner, acyl chain pocket, we constructed a structural homology model using the published crystal structure of *E. coli* ACP bound to a C10 fatty acyl chain (2FAE) as a template (Fig. 2). We constructed a number of single amino acid mutants by exchanging small hydrophobic side-chain residues, such as isoleucine or leucine, with bulkier hydrophobic side-chains such as phenylalanine, methionine, tyrosine, or tryptophan. ACPs initially fold into an inactive apo state. Conversion to the active holo state is achieved through post-translational modification whereby 4′-phosphopantetheine is transferred from co-enzyme A (CoA) to a specific serine residue on the apo-ACP (Ser39 on *S. elongatus* ACP) [8, 16]. Acyl carrier protein over-expression may reduce the CoA pool and lead to toxic accumulation of apo-ACP, which inhibits sn-glycerol-3-phosphate acyltransferase [16, 17], so as a quick check for functional expression of recombinant ACPs, we measured culture growth kinetics over 15 h. Compared to controls, cells over-expressing wild-type (‘WT’) *E. coli* ACP (Ec-ACP), WT *S. elongatus* ACP (Se-ACP), or mutant Se-ACPs all showed suppressed growth at low levels of induction and worsened at higher induction levels (Additional file 1: Figure S1; Additional file 2: Figure S2), suggesting that these recombinant cyanobacterial ACPs were expressed and properly folded.

**Fig. 3:**
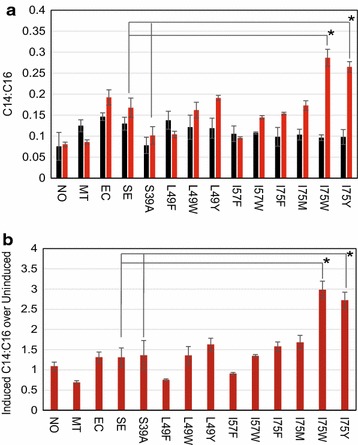
GC–MS analysis of cellular lipids in single ACP mutants. **a** Ratios of C14–C16 molar concentrations for uninduced (*black*) and induced (*red*) strains: no vector (NO), empty vector (MT), WT *E. coli* ACP (EC), WT *S. elongatus* ACP (SE). **b** Fold changes of induced vs. uninduced C14:C16 ratios. The I75 W and I75Y mutants have significantly increased C14:C16 ratios as compared to expressing WT Se-ACP (*p < 0.05, two-tailed student-*t* test). *Data* represent triplicate biological measurements. *Error bars* are standard error of the mean (S.E.M)

To analyze the effect of mutant Se-ACPs on lipid pools, we used gas chromatography–mass spec (GC–MS) to characterize fatty acid methyl esters (FAMEs) derived from lipid pools in Se-ACP over-expressing cells. We compared ratios of FAME peak areas for each sample to minimize effects of differences in growth and sample extraction. We detected peaks for FAMEs derived from the naturally most abundant palmitic acid (C16) and the shorter, less abundant myristic acid (C14) and quantified these peaks in all sample spectra and calibrated to molar concentrations based on a standard curve (Additional file 3: Figure S3). Together, C14 and C16 accounted for >90 % of total fatty acids extracted in all samples (Additional file 4: Figure S4; Additional file 5: Figure S5). The concentration ratios of C14–C16 were calculated and compared across controls and cells expressing Se-ACP point mutants. For all uninduced samples, the C14:C16 ratio was around 0.1 (Fig. 3a). After induction, only the I75 W and I75Y Se-ACP mutants demonstrated a statistically significant increase in the C14:C16 ratio relative to cells expressing WT Se-ACP: the mutants, respectively, caused 3- and 2.7-fold increases (*p* < 0.05, two-tailed student-t test; Fig. 3b), indicating that their lipid pools had shifted toward shorter acyl chains. Mutants that replaced Leu49 or Ile57 did not increase the proportions of shorter fatty acids compared to over-expressing WT ACPs. The side chain of isoleucine 75 is positioned in the hydrophobic pocket close to the terminus of the acyl chain, more so than residues 49 and 57, which contact the side of the acyl chain (Fig. 2a) [12]. Mutating Ile75 to phenylalanine or methionine may cause slight shifts in lipid pool chain-length composition (Fig. 3). Homology modeling indicated that the Tyr75 and Trp75 side-chains protrude roughly two carbon–carbon bond distances further into the hydrophobic acyl chain pocket than an isoleucine at this position (Fig. 2b, c; only I75 W shown). Therefore, I75 W and I75Y Se-ACP mutants may directly hinder elongation from C14 to C16 in fatty acid synthesis and skew the fatty acid pool toward shorter chain lengths.

To explore the potential to further skew cellular lipids toward short-chain lengths, particularly those shorter than 14 carbons long, we introduced secondary point-mutations in addition to the Se-ACP I75 W or I75Y mutations. Amino acids with small hydrophobic side-chains such as isoleucine, valine, or alanine were exchanged for a bulkier methionine, a polar glutamine, or a hydrophilic arginine. Double mutant Se-ACPs did not significantly increase the C14:C16 ratio beyond either single I75 W or I75Y mutation alone (Additional file 3: Figure S3), and did not cause observable production of chains shorter than C14.

As an additional control, the Se-ACP serine 39 residue, which is post-translationally modified with 4-phosphopantetheine, was mutated to alanine (S39A), thereby generating an inactive, obligate apo-ACP. Over-expressing this inactive ACP resulted in similarly low C14:C16 ratio compared to WT (Fig. 3). Growth was suppressed by over-expressing this mutant protein, suggesting that the protein was correctly folded [16, 17].

**Fig. 4:**
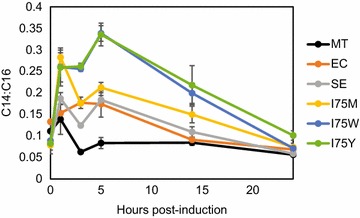
Time Course of C14:C16 Ratios Se-ACP I75 W and I75Y demonstrating the highest C14:C16 cellular lipid ratio at 5 h after induction during the growth phase. As the cell cultures saturate past 14 h, the ratios decrease to the baseline of around 0.05–0.1. Data represent triplicate biological measurements. *Error bars* are S.E.M

These results indicated that expression of mutant ACPs could be used to enhance production of a medium-chain fatty acid. To explore conditions for optimal production, we characterized C14:C16 ratios over a 24-h time course. The lipid pool composition shows that the highest C14:C16 ratio occurs around 5-h post-induction (Fig. 4). Longer induction times resulted in a decreased C14:C16 ratio for all strains, particularly for Se-ACP I75 W and I75Y mutants, which fell and became indistinguishable from controls by 24 h. This highlights the importance of growth phase on lipid composition. During exponential growth, when cells are actively dividing and building new membranes, fatty acid metabolism is highly active, and an abundance of mutated ACPs with reduced pocket sizes likely biases the fatty acid pool toward shorter acyl chains [18]. It may be that membrane synthesis proceeds with greater fidelity as cell growth slows. Alternatively, short-chain fatty acids may be actively replaced with fatty acids of the correct length, which would be more apparent in stationary phase when new C14 fatty acids are not being added to membrane lipids.

**Fig. 5:**
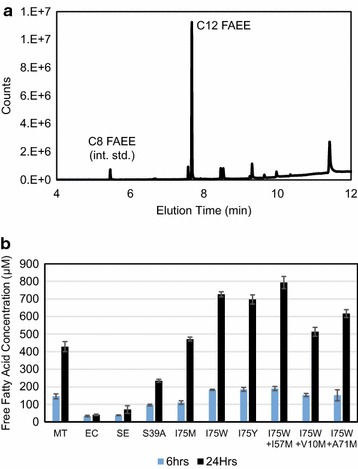
Free fatty acid production by C12 thioesterase. **a** Representative GC-MS trace of FAEEs derived from *cell cultures* shows thioesterase specificity toward 12-carbon acyl chains. **b** FFA concentrations measured from cell cultures at 6 h (*blue*) and 24 h (*black*) post-induction of both the C12 thioesterase and the indicated ACP. The Se-ACP I75 W and I75Y mutants and their derivatives yield more FFA than controls. *Data* represent triplicate biological measurements. *Error bars* are S.E.M

We next tested the effect of mutant ACPs on production of lauric acid (C12). A thioesterase that specifically produces 12-carbon chains (*UcFatB2* from *Cuphea palustris*) [6] was co-expressed with wild-type and mutant *Se*-ACPs, and FFA production was measured by GC–MS analysis of fatty acid ethyl esters (FAEE) derived from the produced FFAs (Fig. 5). We hypothesized that increased levels of shorter chain acyl-ACPs would serve as substrates to the medium chain-specific thioesterase and further increase the yield of medium chain FFAs. In conjunction with expressing the C12 thioesterase, strains over-expressing I75 W or I75Y mutant ACPs significantly increased medium chain FFA yields (Fig. 5); all controls produced less FFA than the I75 W or I75Y mutants. (There were significant differences between the various controls, presumably reflecting the fact that overproducing various forms of ACP can affect fatty acid metabolism by, for example, depleting CoA or non-productively interacting with other enzymes [16, 17]). Combining mutations did not further enhance FFA production (Additional file 6: Figure S6). In addition, FFA yields were uncorrelated to differences in growth rates among all the strains (Additional file 7: Figure S7) and were not affected by beta oxidation knock out (Additional file 8: Figure S8).

## Conclusions

In sum, we have shown that ACP, an essential protein in fatty acid metabolism, can be modified by site-directed mutagenesis to skew cellular lipid pools toward smaller acyl chain lengths. Specifically, expressing certain mutant ACPs enhanced the level of C14 fatty acids in membrane lipids, and by co-expressing mutant ACPs with a chain-length specific thioesterase production of a medium-chain free fatty acid (lauric acid) was enhanced. These results are consistent with a hypothesis that bacterial ACPs influence lipid chain-length during fatty acid synthesis. Other enzymes involved in fatty acid synthesis also likely affect chain-length, and engineering modified acyl chain specificity has been similarly achieved. For example, FabB and FabF catalyze elongation of fatty acid chains (Fig. 1), and have a clearly defined pocket that should accommodate carbon chains up to about 18 [19]. Val et al. engineered the FabF pocket to accommodate a maximum of six carbons [20]. Similarly, the cyanobacterial aldehyde decarbonylase solved structure [21, 22] contains electron density corresponding to a C18 fatty acid or aldehyde; Khara et al. modified this enzyme to have specificity for medium-chain substrates [22]. The C8-, C12-, and C14-specific plant-derived acyl-ACP thioesterases apparently also control length of fatty acid products, although the underlying structural mechanisms have not been identified. Since FFAs contain the hydrophilic carboxylic acid functional group, they are not ideal fuel molecules. Instead, FFAs can act as precursors to further enzymatic modification for transformation into highly desired fuel molecules such as fatty alcohols and alkanes. Engineering such enzymes (e.g., aldehyde decarbonylases, acyl-ACP reductases, and carboxylic acid reductases) toward shorter carbon chain substrate recognition will likely be key to tailoring biofuel formulations. To achieve the ultimate goal of efficient biofuel synthesis, it may be necessary to engineer the length specificity of several enzymes—most such enzymes have evolved to handle chains of 16–18 carbons, but shorter chains are desired in fuels. This technology could help to optimize biofuel yield and molecular makeup, which would benefit the goal of developing energy sources alternative to fossil fuels.

## Methods

### Homology modeling

The structural model of *Se*-ACP harboring a decanoyl-chain was obtained by homology to the published x-ray crystal structure of the *E. coli* decanoyl-ACP (2FAE) using SWISS-MODEL [12].

### Strain construction

Double-stranded DNA encoding *E. coli* and *S. elongatus* ACP genes were synthesized as gBlocks (Integrated DNA Technologies) and cloned into the pCDF-Duet vector by Gibson Assembly [23]. Single- and double-amino acid mutations of the *Se*-ACP gene were incorporated during DNA synthesis. An empty pCDF-Duet-1 vector (Millipore) without the ACP gene was included as control. Plasmids were sequence-verified and transformed into *E. coli* BL21(DE3). For FFA production, the C12 thioesterase gene (*UcFatB2* from *C. palustris*) was cloned into pET-Duet-1 vector (Millipore) and transformed into strains harboring the plasmids carrying the ACP variants.

### Growth kinetics assay

ACP expressing strains in triplicates were inoculated from single colonies representing independent transformants into LB medium, grown overnight to saturation, and back-diluted into M9 minimal media containing 0.4 % glucose. The cultures were grown to mid-exponential phase (OD ~0.4), dispersed into 96-well plates, induced with various concentrations of IPTG, and left to grow shaking at 37 °C in a plate reader (BioTek NEO). The optical densities (OD) of the cultures were recorded every 5 min over 15 h by the plate reader. The growth curves, as well as the final OD after 15 h were compared among the strains to quantify growth suppression by ACP over-expression.

### Analysis of cellular lipid composition

ACP expressing strains in triplicates were inoculated in LB, grown overnight, and back-diluted into M9 minimal media containing 3 % glucose. The cultures were grown to an optical density of 0.4, induced with 1 mM IPTG, and grown for six more hours at 37 °C. For the time course experiment (Fig. 4), the cultures were left to grow for up to 24 h. After growth, 10 ml of cell culture was used for extraction and analysis, corresponding to wet biomass weights (pellet) of around 5 mg (ACP over-expressing, growth defect) to 10 mg (not inducing ACP). The cells were pelleted and resuspended in 1:1 methanol:chloroform with 2 % glacial acetic acid for lysis, hydrolysis of membrane lipids, and solubilization of fatty acids into the organic phase. Octanoate (C8 fatty acid) was added into the mixture as an internal standard. After vigorous mixing by vortexing, the organic phase was transferred by glass pipettes into glass vials, and the chloroform solvent was evaporated by nitrogen. The vials were then treated with methanol containing 1.25 M HCl at 50 °C for 15 h to catalyze methylation of the fatty acids. The reaction was quenched by adding 5 ml of 100 mg/ml sodium bicarbonate. 0.5 ml hexane was added and the mixture was vortexed vigorously before the hexane phase containing the FAME was extracted and subsequently analyzed on a GC–MS (Agilent 6890/5975) [24]. First a standard set of FAMEs with varying chain lengths was run on the GC–MS in scan mode to determine the identity of each fatty acid peak based on the elution time for each fatty acid and comparison of its fragment profile to those in the NIST database (via Agilent ChemStation software). Fatty acid peaks from the extracted cell samples were also identified using scan mode. To quantify peak areas, the background was minimized using Selective Ion Mode (SIM) whereby the elution times were used to determine fatty acid identity and only the most dominant mass peaks pertaining to each fatty acid methyl ester were counted. For calibration of concentrations, standard curves for C14 and C16 FAMEs dissolved in hexane were taken in the range of 0.1–400 mg/L. A linear fit of hexane background-subtracted peak area to known concentration was extracted in the 0.1–6.215 mg/L range to cover the range of concentrations seen in the cell samples. Molar concentration was determined by dividing mass concentration (mg/L) by the molecular weight of C14 FAME (242 g/mol) or C16 FAME (270.4 g/mol). To compare the proportions of different chain lengths in each sample, the molar concentration ratio of C14 to C16 FAME was taken.

### Analysis of free fatty acid (FFA)

ACP and C12 thioesterase-expressing strains in triplicates were grown in M9 minimal media containing 3 % glucose and induced with IPTG as described above. After 6 or 24 h of growth, five microliters of each culture (cells and media, as medium chain FFA may be secreted) were transferred to wells of a new 96-well plate for high-throughput spectrometric determination of FFA concentration using the Roche Free Fatty Acid Kit (Product Number 11383175001). The FFA is first converted via acyl-CoA synthetase into acyl-CoA, which is then oxidized in the presence of acyl-CoA oxidase to enoyl-CoA, releasing H_2_O_2_ in the process that converts 2,4,6-tribromo-3-hydroxy-benzoic acid (TBHB) and 4-aminoantipyrine (4-AA) to a red dye detectable by spectrometer at 546 nm. To specifically detect lauric acid, cultures of ACP plus thioesterase-expressing cells were lysed and extracted with chloroform. The FFA was ethylated and run on the GC–MS to determine the spectrum of chain lengths.

## Additional files

10.1186/s13068-016-0430-4 Growth Suppression by ACP expression. Growth of *E. coli* is suppressed by induction of Se-ACP expression increasing from 0 mM (*black*), 0.1 mM (*blue*) to saturation at 1 mM (*red*) IPTG. The growth defect is likely due to inhibition of phospholipid metabolism by apo-ACP. Se-ACP I75 W expression (**b**) shows similar growth suppression compared to WT (**a**), indicating proper folding and functionality of ACP. All mutant ACPs show similar growth curves (data not shown). Representative growth curves are shown
10.1186/s13068-016-0430-4 Growth Suppression by ACP expression. Shown are changes in culture OD of *E. coli* strains induced to overexpress various ACPs versus their uninduced condition (0 mM IPTG). Culture densities were measured after 15 h of growth in M9 minimal media with 0.4 % glucose. When overexpressed, most mutants show equal or stronger growth suppression vs. WT Se-ACP. Data represent triplicate biological measurements. *Error bars* are S.E.M
10.1186/s13068-016-0430-4 GC–MS Concentration Calibration Curve for C14 and C16 FAMEs The C14-C16 peak areas were quantified as a function of their known concentrations over the wide range from 0.1–400 mg/L (**a**). Linear fits were extracted from the hexane background-subtracted standard curves in the low concentration range to calibrate the concentrations of C14 and C16 from cell samples, which lie in this lower range (**b**). Eventually the calibrated mass concentrations were converted to molar concentrations by dividing by molecular mass
10.1186/s13068-016-0430-4 GC–MS Chromatogram of FAME extracted from E.coli culture FAME peaks were identified by retention time and mass spectrum. C14 and in particular C16 peaks were most dominant in all cultures. C18 peak was much less significant, and fatty acid shorter than C14 were not reliably detected
10.1186/s13068-016-0430-4 GC–MS chromatogram and mass spectra of BL21(DE3) expressing WT *E.coli* ACP Full (A) and zoomed-in (B) chromatogram in mass scan mode highlighting the saturated C14, C16 peaks from the extracted sample and the peak of saturated C15 added to during extraction as internal standard. (C-L) mass spectra of major peaks in the chromatogram, labelled by the peak identity, retention time, and integrated peak area
10.1186/s13068-016-0430-4 GC–MS analysis of cellular lipids from double mutants. Combining the single Se-ACP I75 W or I75Y point mutants with a second set of residues mutated to methionine does not significantly change the C14:C16 ratio from that observed for the single point mutants alone. Mutating this second set of residues to arginine (A71R) or glutamine (A71Q) reduced the C14:C16 ratio to WT Se-ACP levels. *Data* represent triplicate biological measurements. *Error bars* are S.E.M
10.1186/s13068-016-0430-4 Culture OD of strains co-expressing recombinant ACP and C12 thioesterase. OD600 of the cultures were measured in stationary phase using a Biotek NEO plate reader. The mutants that showed largest increase in FFA (I75 W, I75Y) measured higher OD600 compared to the wild type controls (EC, SE), indicating that increased medium chain FFA production is not a consequence of decrease growth rate
10.1186/s13068-016-0430-4 Effect of Eliminating Beta Oxidation by fadD Knock-Out on FFA C12 FFA yields were compared between wild-type BL21(DE3) and fadD knocked-Out (KO) BL21(DE3) strains after 24 h of induced expression of C12 thioesterase (1 % arabinose) and ACP (100uM IPTG), where the ACP vector contained either empty vector (MT), wild-type cyano ACP (SE), or I75Y cyano ACP mutant. The fadD-KO strains that eliminated beta oxidation of FFA did not present increased yields of C12 FFA

## Abbreviations

ACP: acyl carrier protein
FFA: free fatty acid
