# Supplementary figures and images for: Engineering acyl carrier protein to enhance production of shortened fatty acids
Source: Biotechnol Biofuels. 2016 Feb 2;9:24. doi: 10.1186/s13068-016-0430-4 (PMC4736557; doi:10.1186/s13068-016-0430-4)

**A**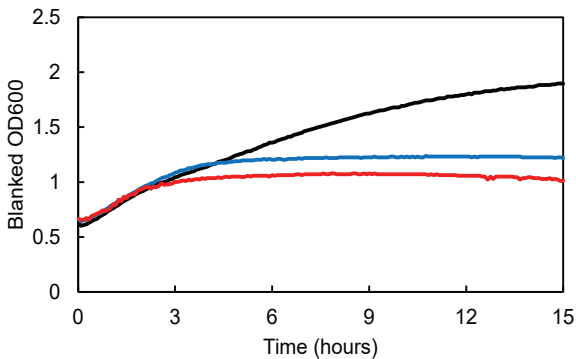**B**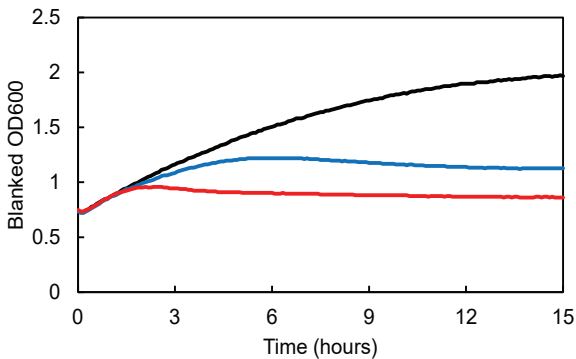

Supplement: Supplementary file 1 — 10.1186/s13068-016-0430-4 Growth Suppression by ACP expression. Growth of E. coli is suppressed by induction of Se-ACP expression increasing from 0 mM (black), 0.1 mM (blue) to saturation at 1 mM (red) IPTG. The growth defect is likely due to inhibition of phospholipid metabolism by apo-ACP. Se-ACP I75 W expression (b) shows similar growth suppression compared to WT (a), indicating proper folding and functionality of ACP. All mutant ACPs show similar growth curves (data not shown). Representative growth curves are shown [file 13068_2016_430_MOESM1_ESM.pdf]

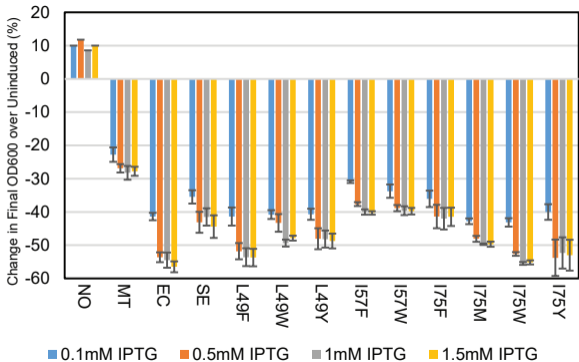

Supplement: Supplementary file 2 — 10.1186/s13068-016-0430-4 Growth Suppression by ACP expression. Shown are changes in culture OD of E. coli strains induced to overexpress various ACPs versus their uninduced condition (0 mM IPTG). Culture densities were measured after 15 h of growth in M9 minimal media with 0.4 % glucose. When overexpressed, most mutants show equal or stronger growth suppression vs. WT Se-ACP. Data represent triplicate biological measurements. Error bars are S.E.M [file 13068_2016_430_MOESM2_ESM.pdf]

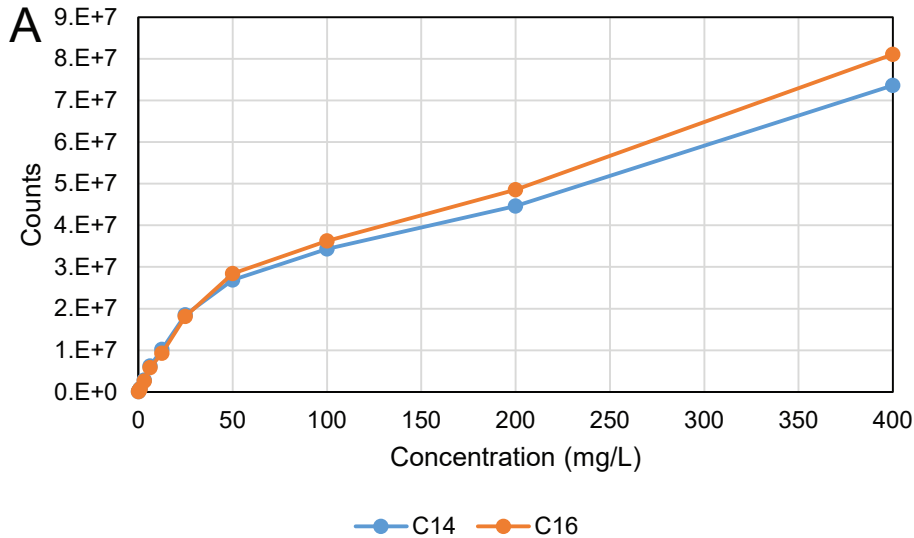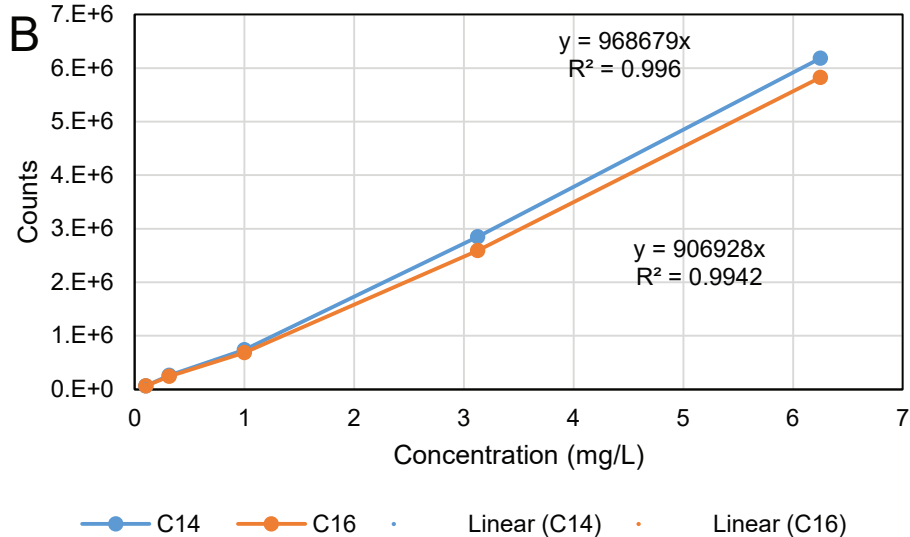

Supplement: Supplementary file 3 — 10.1186/s13068-016-0430-4 GC–MS Concentration Calibration Curve for C14 and C16 FAMEs The C14-C16 peak areas were quantified as a function of their known concentrations over the wide range from 0.1–400 mg/L (a). Linear fits were extracted from the hexane background-subtracted standard curves in the low concentration range to calibrate the concentrations of C14 and C16 from cell samples, which lie in this lower range (b). Eventually the calibrated mass concentrations were converted to molar concentrations by dividing by molecular mass [file 13068_2016_430_MOESM3_ESM.pdf]

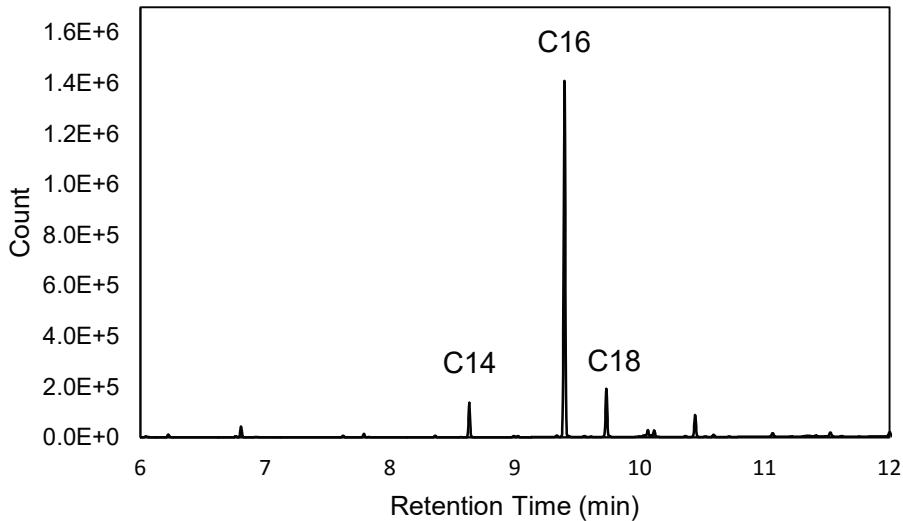

Supplement: Supplementary file 4 — 10.1186/s13068-016-0430-4 GC–MS Chromatogram of FAME extracted from E.coli culture FAME peaks were identified by retention time and mass spectrum. C14 and in particular C16 peaks were most dominant in all cultures. C18 peak was much less significant, and fatty acid shorter than C14 were not reliably detected [file 13068_2016_430_MOESM4_ESM.pdf]

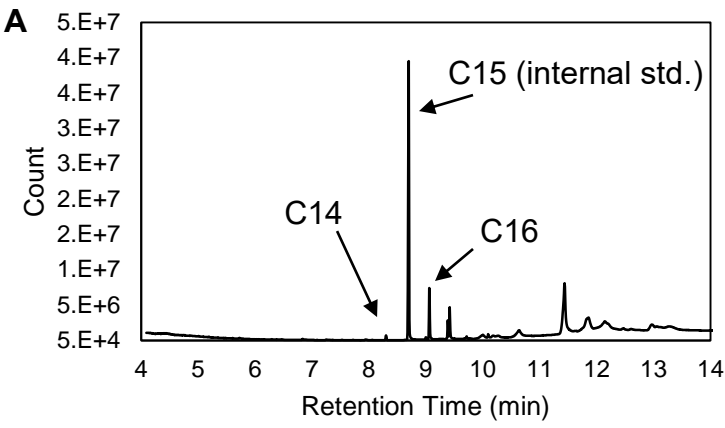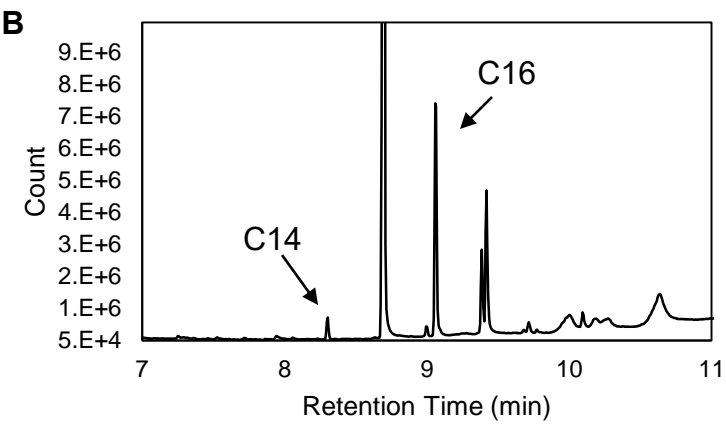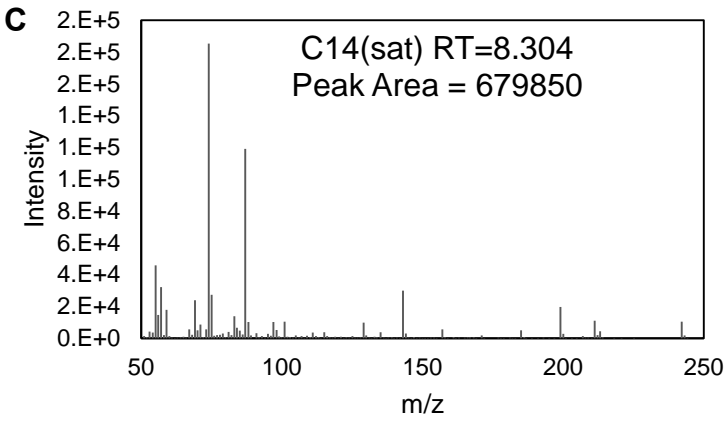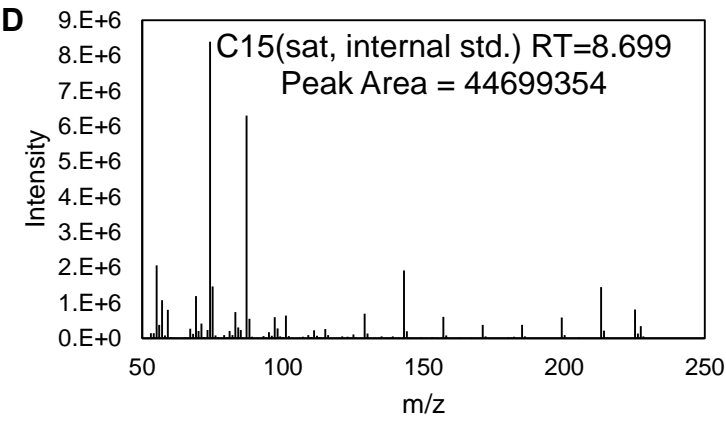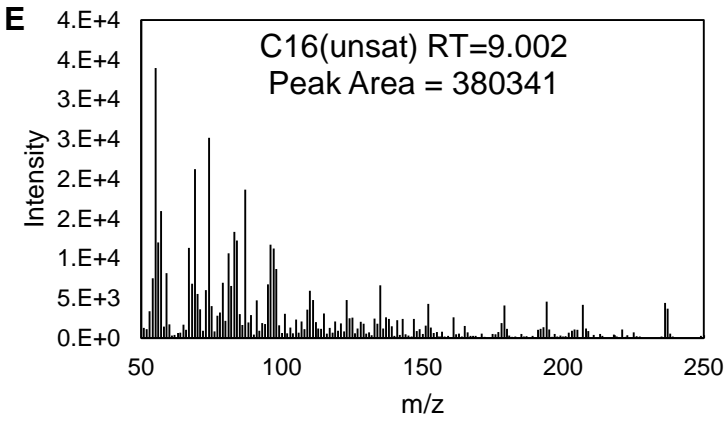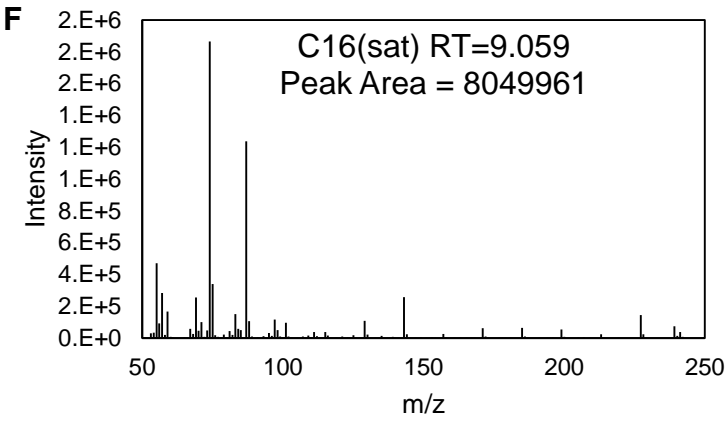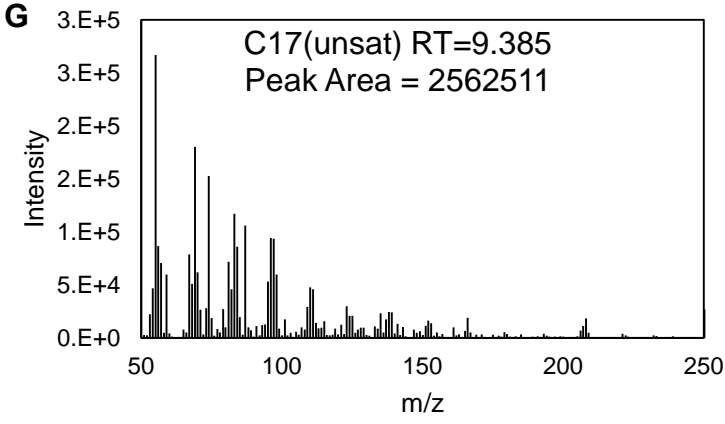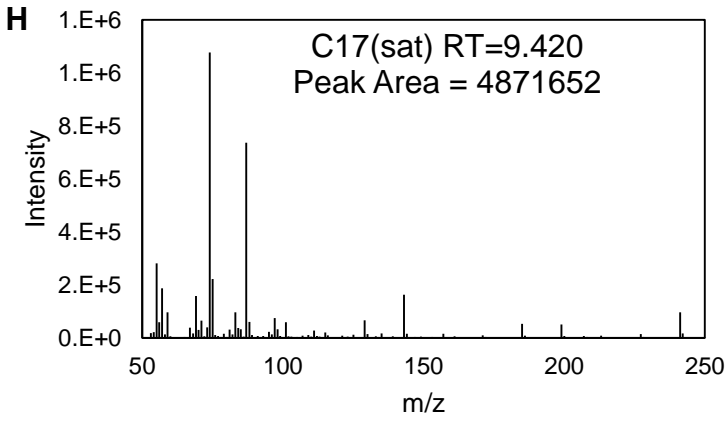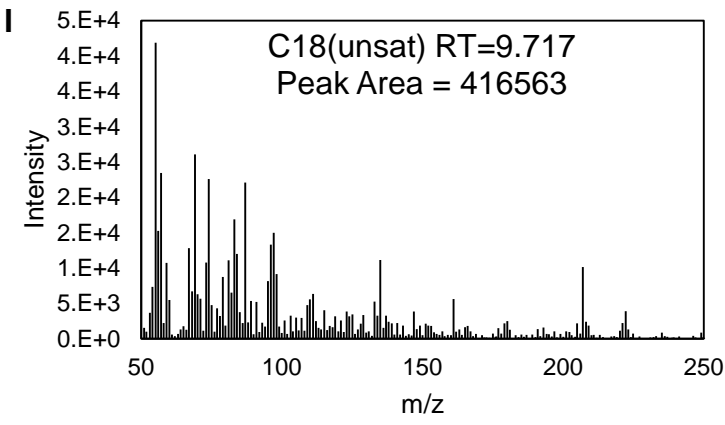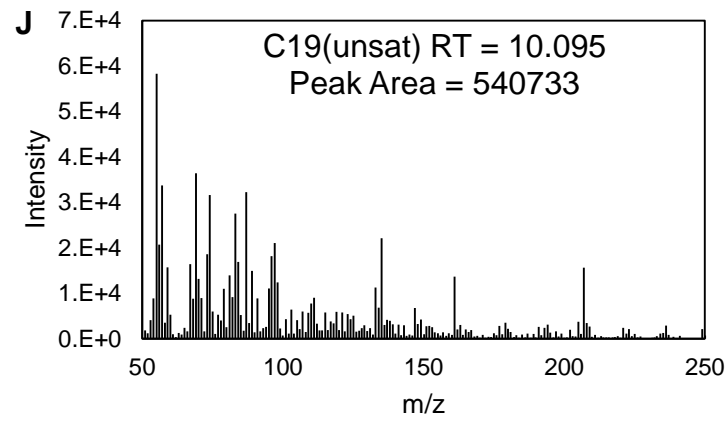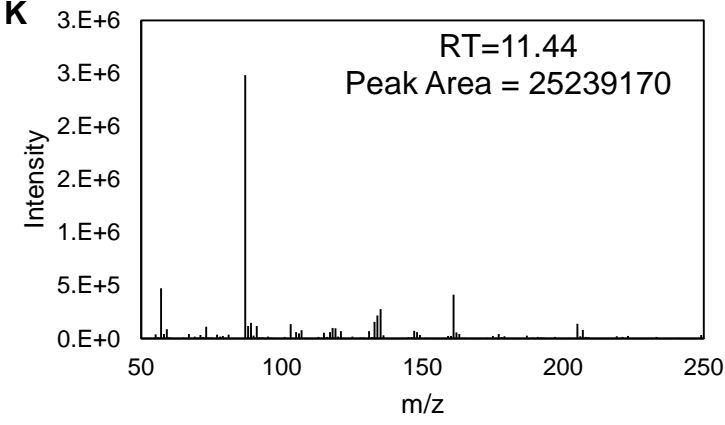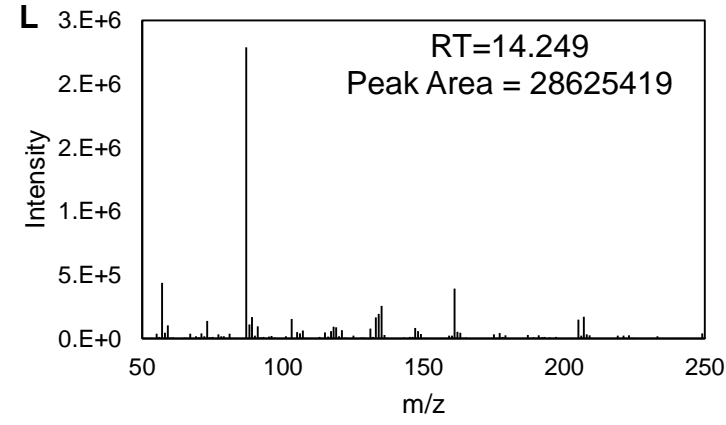

Supplement: Supplementary file 5 — 10.1186/s13068-016-0430-4 GC–MS chromatogram and mass spectra of BL21(DE3) expressing WT E.coli ACP Full (A) and zoomed-in (B) chromatogram in mass scan mode highlighting the saturated C14, C16 peaks from the extracted sample and the peak of saturated C15 added to during extraction as internal standard. (C-L) mass spectra of major peaks in the chromatogram, labelled by the peak identity, retention time, and integrated peak area [file 13068_2016_430_MOESM5_ESM.pdf]

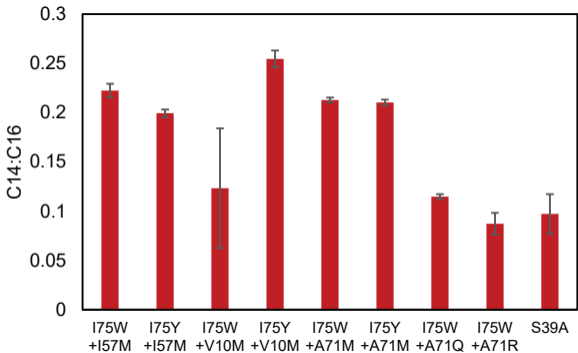

Supplement: Supplementary file 6 — 10.1186/s13068-016-0430-4 GC–MS analysis of cellular lipids from double mutants. Combining the single Se-ACP I75 W or I75Y point mutants with a second set of residues mutated to methionine does not significantly change the C14:C16 ratio from that observed for the single point mutants alone. Mutating this second set of residues to arginine (A71R) or glutamine (A71Q) reduced the C14:C16 ratio to WT Se-ACP levels. Data represent triplicate biological measurements. Error bars are S.E.M [file 13068_2016_430_MOESM6_ESM.pdf]

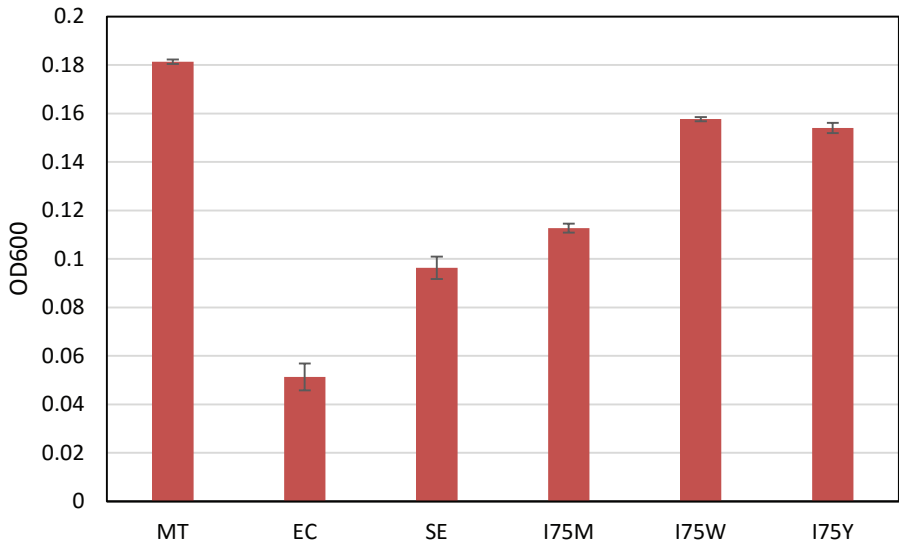

Supplement: Supplementary file 7 — 10.1186/s13068-016-0430-4 Culture OD of strains co-expressing recombinant ACP and C12 thioesterase. OD600 of the cultures were measured in stationary phase using a Biotek NEO plate reader. The mutants that showed largest increase in FFA (I75 W, I75Y) measured higher OD600 compared to the wild type controls (EC, SE), indicating that increased medium chain FFA production is not a consequence of decrease growth rate [file 13068_2016_430_MOESM7_ESM.pdf]

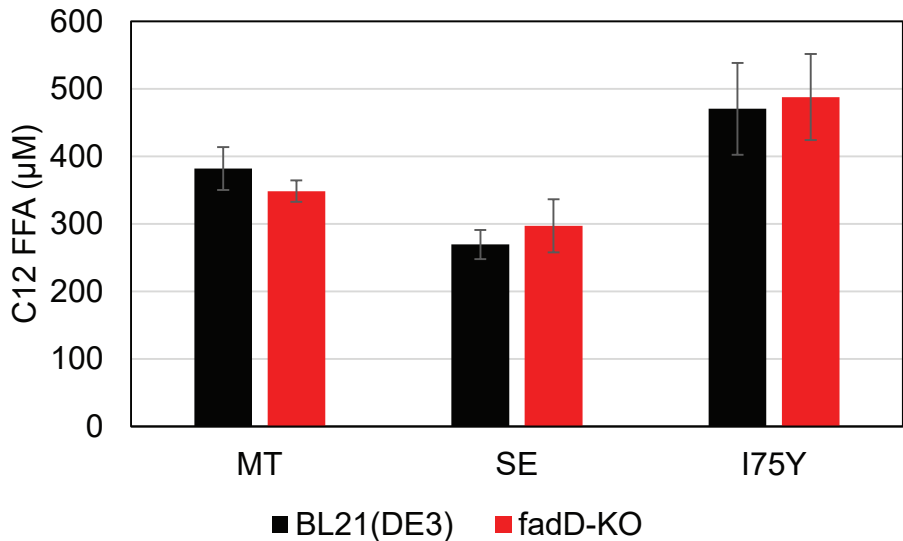

Supplement: Supplementary file 8 — 10.1186/s13068-016-0430-4 Effect of Eliminating Beta Oxidation by fadD Knock-Out on FFA C12 FFA yields were compared between wild-type BL21(DE3) and fadD knocked-Out (KO) BL21(DE3) strains after 24 h of induced expression of C12 thioesterase (1 % arabinose) and ACP (100uM IPTG), where the ACP vector contained either empty vector (MT), wild-type cyano ACP (SE), or I75Y cyano ACP mutant. The fadD-KO strains that eliminated beta oxidation of FFA did not present increased yields of C12 FFA [file 13068_2016_430_MOESM8_ESM.pdf]
